# Field Diarrhoea

**Published:** 1915-11

**Authors:** 


**Field Diarrhoea.** Ad. Schmidt, Munch. Med. Woch. No. 36, 1914. Multivalent serum is advised, as few cases are due to pure infection with either Kruse-Shiga, Flexner or Y-Strong types of dysentery bacilli, intermediate types and mixed strains being far more common. 3 doses of 10 c.c., in 2 or 3 days, usually prove curative. Kaolin, 300 c.c. of decoction of simaruba, made in 20% strength and diluted to 10% are of value but the latter tastes badly and may irritate. Emetin he does not consider of value in bacillary cases.

